# Cultivating the uncultured: growing the recalcitrant cluster-2 *Frankia* strains

**DOI:** 10.1038/srep13112

**Published:** 2015-08-19

**Authors:** Maher Gtari, Faten Ghodhbane-Gtari, Imen Nouioui, Amir Ktari, Karima Hezbri, Wajdi Mimouni, Imed Sbissi, Amani Ayari, Takashi Yamanaka, Philippe Normand, Louis S Tisa, Abdellatif Boudabous

**Affiliations:** 1 Laboratoire Microorganismes et Biomolécules Actives, Université de Tunis El Manar (FST) & Université Carthage (INSAT), 2092, Tunis, Tunisia; 2 Forest and Forestry Products Research Institute, Ibaraki 305-8687, Japan; 3 Ecologie microbienne, UMR CNRS 5557, Université Lyon1, Université de Lyon, 69622 cedex, Villeurbanne, France; 4 Department of Molecular, Cellular and Biomedical Sciences, University of New Hampshire, Durham, NH 03824, USA

## Abstract

The repeated failures reported in cultivating some microbial lineages are a major challenge in microbial ecology and probably linked, in the case of *Frankia* microsymbionts to atypical patterns of auxotrophy. Comparative genomics of the so far uncultured cluster-2 *Candidatus* Frankia datiscae Dg1, with cultivated Frankiae has revealed genome reduction, but no obvious physiological impairments. A direct physiological assay on nodule tissues from *Coriaria myrtifolia* infected with a closely-related strain permitted the identification of a requirement for alkaline conditions. A high pH growth medium permitted the recovery of a slow-growing actinobacterium. The strain obtained, called BMG5.1, has short hyphae, produced diazovesicles in nitrogen-free media, and fulfilled Koch’s postulates by inducing effective nodules on axenically grown *Coriaria* spp. and *Datisca glomerata*. Analysis of the draft genome confirmed its close proximity to the *Candidatus* Frankia datiscae Dg1 genome with the absence of 38 genes (trehalose synthase, fumarylacetoacetase, etc) in BMG5.1 and the presence of 77 other genes (CRISPR, lanthionine synthase, glutathione synthetase, catalase, Na+/H+ antiporter, etc) not found in Dg1. A multi-gene phylogeny placed the two cluster-2 strains together at the root of the *Frankia* radiation.

Ever since Robert Koch first proposed a series of postulates to establish a causal link between a microbe and a disease, a basic tenet of microbiology has been the isolation of the causative microbes in pure culture^1^. Because many microbes such as fungi (*Gigaspora, Rhizophagus*) or bacteria (*Mycobacterium leprae, Wolbachia,* etc), cannot be cultivated in pure culture, it is now widely accepted these postulates should not be strictly considered^2^. Nevertheless, growth in pure culture remains an irreplaceable tool to study the physiology of an organism. Reasons behind these failures are varied including fragility of cells buffered within host tissues, cumulative damages by reactive compounds to cellular constituents such as DNA and membranes, or loss of genes resulting in auxotrophy^3^.

*Frankia* is a slow-growing actinobacterium. First described in 1866 as the causative agent of nodules found on the roots of *Alnus* and *Elaeagnus*^4^, it resisted cultivation attempts for a century. Because of its importance in ecological successions, studies were made using crushed nodules. However, the lack of isolates caused erroneous conclusions on plant host range and phylogenetic affiliation of the microbes present^5^. In 1978, the first *Frankia* isolation was reported^6^ using an enzymatic maceration of host tissues and a complex growth medium, and has led to many subsequent successful reports on the isolation of strains from the different host plants. Molecular phylogenetic approaches using both cultured and uncultured bacteria have grouped *Frankia* into 4 clusters^7^ and 3 of these clusters now routinely yield isolates from most provenances. However in spite of repeated attempts in numerous laboratories, cluster-2 strains that nodulate Rosaceae, Coriariaceae, Datiscaceae and *Ceanothus* have consistently resisted isolation. Nevertheless, the genome of a cluster-2 strain was determined by directly sequencing DNA isolated from nodule tissues^8^ and a comprehensive study of its metabolic pathways permitted to conclude there was no missing function that would impede cluster-2 strains to grow in pure culture^9^.

One of the aims of the cultivation of a microbe is to provide knowledge on its physiological properties, but this information is also necessary to tailor the growth media for isolation of this given microbe. This circular logic is usually broken through trials and errors. The Biolog (Hayward CA) assay, which allows the assessment of physiological properties of cells, does not depend on growth, but rather on the ability of cells to reduce an indicator dye, tetrazolium to the purple insoluble formazan^10^. We decided to use this system to study the physiology of cluster-2 *Frankia* strains and thus adapt our isolation conditions to fit the endophytic cells.

The genome of *Candidatus* Frankia datiscae Dg1, an uncultured cluster-2 strain, was analyzed to provide information on these symbionts for their isolation. Although no metabolic pathways appeared to be incomplete, the genome contained a reduced number of genes involved in stress responses^8^. With this information, a high-throughput microplate assay was used to first assess the viability of the microsymbiont. These live/dead assays were performed under different pH conditions, with various carbon sources and in the presence or absence of oxygen scavengers and osmolytes. Strikingly, the viability of these endophytic cell clusters was highly sensitive to pH. Viable endophytic cells were recoverable only from PM10 microplate wells with a pH higher than 8.5 for up to two months as shown by the live/dead assay. Therefore, buffers used to release vesicle clusters from root nodules and to inoculate Biolog microplates were adjusted to pH 9 for all subsequent experiments, which permitted a substantial extension of cell endophyte viability in numerous wells in PM1 and PM2A microplates. Pyruvate, propionate, succinate, and acetate are all generally used to cultivate other *Frankia* strains^11^,^12^,^13^, and were identified as potential carbon and energy sources for the isolation medium.

Based on this information, a modified BAP medium^13^,^14^ buffered to pH 9 and supplemented with several organic acids to replace the usual propionate as carbon source and several oxygen scavengers was used, and yielded very slow-growing brownish colonies. These colonies required a 14 month incubation period to yield sufficient material for subculturing. One colony, termed BMG 5.1, was selected, and gently fragmented for propagation. The isolate was maintained in pure culture and found to be free of contaminants after plating a sample of it on solid LB medium. The isolate grew slowly, forming hard-to-fragment colonies in liquid medium (Fig. 1A). Under phase contrast microscopy, the isolate was observed to consist of a mycelial mat containing short irregular filaments (Fig. 1B) with thick elbow-like regions that could be due to discontinuous growth (Fig. 1C). Globular diazovesicles, the site of nitrogen fixation in *Frankia* under symbiotic and pure-culture conditions^15^, were produced in nitrogen-deficient (BAP medium without NH_4_Cl) medium (Fig. 1D). No sporangia were observed under the conditions tested. The culture grown on nitrogen-deficient medium was also able to reduce acetylene (135 +/−11 nmoles h-1 mg-1 protein), a proxy for nitrogen-reduction, as soon as vesicles were formed. The results from the Biolog assays on isolate BMG5.1 grown in pure culture (Table S1) showed a similar pattern of physiological properties as predicted from the viability screening of microsymbiont clusters. Furthermore, isolate BMG5.1 showed an optimum growth yield at pH 9.5 (Fig. 2). In contrast, the other *Frankia* strains showed optimum growth yield around pH 7-7.5. Isolate BMG5.1 failed to grow in medium at pH 6.5 that was usually used for other *Frankia* isolates^14^.

Phylogenetic analysis shows that BMG5.1 strain belongs to *Frankia* cluster-2 and shares 99.6%, 98.16%, 98.76% and 99.4% identity with the *Candidatus* Frankia datiscae Dg1, respectively on the basis of 16S rRNA, *nif*H, *gyr*B and *gln*A gene sequences. A multigene analysis using concatenated *atp*I, *gyr*A, *fts*Z, *sec*A and *dna*K genes confirms the close proximity of the new isolate with *Candidatus* Frankia datiscae Dg1 at the root of the genus radiation (Fig. 3).

The draft genome of *Frankia* sp. strain BMG5.1 was determined and found to consist of 5,795,263 bp with a 71% G+C and 5,245 predicted protein-coding genes, characteristics which are very close to those of the *Candidatus* Frankia datiscae Dg1 genome (Table 1). All of the generated 500000 reads were found to be *Frankia* sequences indicating the absence of potential contaminants. Comparative analysis showed that the two genomes had an average nucleotide identity (ANI) of 96.93%, which would correspond to the same species^16^ and ANI scores of 82.32, 82.97, 83.05 and 83.68% with *Frankia* sp. strains EuI1c, ACN14a, EAN1pec and CcI3 genomes, respectively^17^. Several genes were found to be present in BMG5.1, but were absent in Dg1 (Table S2) including catalase, choline dehydrogenase and glutathione synthase that would correspond to a higher level of resistance to *in vitro* culture conditions for BMG5.1. Interestingly, the *Frankia* sp. BMG5.1 genome contains multiple Na+/H+ antiporter genes (Fig. S1).

To fulfill Koch’s postulates, plant infectivity experiments were performed on seedlings of *Coriaria japonica*, *Coriaria myrtifolia* and *Datisca glomerata,* all of which are hosts of cluster-2 *Frankia*. The seedlings were grown axenically and transferred to growth pouches prior to inoculation with the isolate. After inoculation with isolate BMG5.1, the plants produced root nodules within 30–35 days. These nodules were actively fixing nitrogen as determined by the better growth of inoculated seedlings in N-free medium relative to non-inoculated controls (Fig. 4) and by the result of the acetylene reduction assay^18^ (211 + 3 nmoles h^−1^ mg^−1^ of nodule). The presence of BMG5.1 was confirmed by extracting DNA from the plant root nodules, amplifying and sequencing of the four DNA markers (16S rRNA, *nif*H, *gyr*B and *gln*A), which yielded identical sequences to the BMG5.1 isolate. Furthermore, BMG5.1 was reisolated from these nodules using the same isolation methods, grown in pure culture, and shown by PCR-sequencing to have identical markers (16S rRNA, *nif*H, *gyr*B and *gln*A).

It is a stressful situation for a bacterium buffered by host tissues and under oxygen levels kept low by neighboring mitochondria^19^ to grow on synthetic medium. Down-regulation of many stress proteins in symbiotic *Frankia alni*^20^ also indicates that symbiosis may be less stressful than saprotrophic growth. In the case of alders, defense peptides have been shown to increase *Frankia* porosity^21^ and would render symbiotic cells particularly fragile. Thus, if *in vitro* growth conditions greatly differed from those found in the host tissues, isolation would be extremely difficult and would probably not occur. This is the reason why it is necessary to modify growth media to make them as comparable to the physiological conditions existing inside host tissues as possible. In the case of cluster-2 *Frankia,* these changes to the growth medium included the addition of oxygen scavengers and the use of an alkaline pH. Physiological assessment of uncultivated cells can thus provide reliable physiological data, leading to testable hypotheses for pure culture conditions.

This new *Frankia* isolate may form, together with *Candidatus* Frankia datiscae Dg1 strain, a different species that is mildly alkaliphilic. A number of alkaliphiles have been found among various lineages, many of which are actinobacteria in arid soils^22^. Adaptation to an alkaline biotope occurs through either local acidification of the medium or modification of the wall. Among the genes present in the BMG5.1 genome but absent in Dg1 were the Na+/H+ antiporters, which have been described as permitting cells to adjust to high pH^22^. A pure culture of *Frankia* BMG5.1 should be helpful in experiments directed toward our understanding this requirement for these unusual physiological conditions.

Clearly, the use of bioinformatics analysis of the genome and physiological bioassays has resulted in the isolation of a previously recalcitrant microbe. This dual approach could be extended to other microbes considered as yet uncultivable such as *Mycobacterium leprae* or *Rhizophagus intraradices*, which should markedly facilitate their study and biotechnological uses.

## Methods

### Vesicle clusters viability and growth requirement screening

Root nodules obtained from *Coria-ria japonica*^23^ growing in Japan (Tosa district) were used to inoculate *Coriaria myrtifolia* seedlings grown on sterile sand and maintained in a greenhouse. Root nodules were harvested, surface disinfected (Na hypochlorite 1.05% for 30 min) and washed several times with sterile distilled water. Preparation of vesicle clusters was performed as described previously^24^. Lobes were crushed in 0.05 M sodium phosphate buffer, pH 7.2 using a Broeck tissue homogenizer and incubated with the following scavengers for hydrogen peroxide (10 mM N,N’-dimethylthiourea) hydroxyl radical (0.28 M dimethylsulfoxide), for singlet oxygen (0.02 M L-histidine), for superoxide anion (10 mM 4,5-dihydroxybenzene-1,3-disulfonate), for peroxyl radical (100 μM α-tocopherol) and for peroxynitrite anion (100 μM uric acid). Released microsymbiont vesicle clusters were retained by gravity filtration on the 52-μm nylon screen and used to assess cells viability using PM1 and PM2A microplates (Biolog) for carbon sources, as well as PM9 and PM10 microplates (Biolog) for pH and NaCl tolerance, respectively. Viability of endophytic cell clusters was weekly assessed in a subset of PM1, PM2A, PM9 and PM10 microplates using only the live/dead assay BacLight Bacterial Viability Kit for microscopy (Life Technologies, Paisley, UK).

### Growing Coriaria endophyte

Vesicle clusters of *Coriaria* endophyte were prepared as described above except that sodium phosphate buffer was adjusted to pH9. BAP medium^14^ containing in mM: K_2_HPO_4_, 4; KH_2_PO_4_, 7; MgSO_4_, 0.1; CaCl_2_, 0.07; FeNaEDTA, 10 mg/l and Hoagland’s microelements^25^ with 5 mM NH_4_Cl (BAP+) was supplemented with selected carbon sources, each added to a final concentration of 10 mM; pyruvate, propionate, succinate and acetate and with a mixed vitamin stock solution contained the following (per liter): 4-aminobenzoic, acid, 40 mg; D-(+)-biotin, 10 mg; nicotinic acid, 100 mg; calcium D(+) −pantothenate, 50 mg; pyridoxamine dihydrochloride, 100 mg; and thiamine dihydrochloride, 100 mg was inoculated with clumps of microsymbiont vesicle clusters in 10 ml glass tubes containing 3 ml liquid medium adjusted to pH9 using TRIS (tris-hydroxy-methyl-aminomethane) and incubated in the dark at 25 °C without agitation. Subcultures of isolated bacteria were characterized by 16S rRNA PCR amplification and sequencing using primers S-D-Bact0008-a-S-20 and S-D-Bact-1495-a-A-20^26^.

### Host plant infectivity and molecular identification

Surface sterilized seeds of *C. japonica, C. myrtifolia* and *D. glomerata* were germinated on moistened filter paper in Petri dishes, at 20 °C under daylight illumination and transferred to sterile moist sand. Three week old seedlings were inoculated by applying the equivalent to 10 μg of protein of washed cultures of the isolate previously syringed with a 21G sterile needle. Plantlets were checked for nodules every week. After 9 weeks, nodules were harvested and used for endophyte reisolation and direct DNA extraction. DNA was extracted from root nodules and isolates using Plant DNeasy kits (Qiagen, Hilden, Germany) and characterized by PCR/sequencing of 16S rRNA , *nif*H, *gyr*B and *gln*A genes using respectively S-D-Bact0008-a-S-20 and S-D-Bact-1495-a-A-20^26^, nifHF and nifHR^27^, FragyrBF and FragyrBR^28^ and DB41 and DB44^29^. The generated sequences were then analyzed by BLASTN^30^ and compared to those obtained for BMG5.1.

### Acetylene reduction activity

*C. japonica* and *D. glomerata* seedlings with and without nodules were assayed for nitrogenase activity by monitoring acetylene reduction^18^ . About 1g of nodules was harvested and placed in sterile Vacutainer (Beckton-Dickinson, Franklin Lakes, NJ) tubes which had 10% of the gas volume replaced with acetylene. After a 60 min incubation at 30 °C, 1 mL of the gas volume was injected into a Girdel gas chromatograph (Suresnes, France) equipped with a flame ionization detector.

A similar assay was made for a BMG5.1 culture in BAP medium without ammonium (BAP-). Vacutainer tubes (10 mL) were inoculated with 5 ml of the liquid cultures and after 1 week, 10% (vol/vol) of the remaining gas volume was replaced with acetylene and incubated for 60 min at 30 °C.

### Biolog and growth at different pH

*Frankia* BMG5.1 cells were harvested by centrifugation (9000 x g, 15 min), washed three time and incubated four days in BAP- mineral solution (no carbon source) to deplete nutritional reserves. The cells were homogenized by repeated passages through a 25G needle. Biolog microplates were inoculated with cells at 10% transmittance suspended in IF0 buffer (supplied by the manufacturer) adjusted to pH 9 for PM1, PM2A and PM9 microplates and supplemented with pyruvate (10 mM final) for PM9 and PM10 microplates and incubated at 28 °C for 20 days. The new isolate BMG5.1 and strains representing the three other *Frankia* clusters; BMG5.12 (cluster-3), ACN14a (cluster-1a), CcI3 (cluster-1b) and EuI1c (cluster-4) were grown in BAP+ media adjusted to different pH. Organic buffers used to reach the desired pH were MES (2-N-morpholinoethanesulfonicacid) for pH 6.5, and MOPS (3-(N-morpholino)-propanesulfonic acid) for the pH range 6.5–7 and TRIS (tris-hydroxy-methyl-aminomethane) for pH above 7. After 3 weeks of growth, growth yield was determined by measuring total cellular protein. After harvesting, bacterial cell samples were suspended in 1.0 N NaOH and solubilized by incubating for 15  min at 90 °C. Total cellular protein levels were measured using the BCA method^31^.

### Genome sequencing of the BMG5.1 strain

Approximately 500 mg of cells obtained from 2 L of 2 months old cultures were washed and their DNA extracted using a Plant DNeasy kit (Qiagen). Highly pure genomic DNA from strain BMG5.1 was used to generate a 300-bp library for paired-end 2 × 100 Illumina sequencing (Imagif, Gif-sur-Yvette, France) from 500000 reads. Sequences assembly was performed using the Velvet de novo assembler^32^. The assembled *Frankia* sp. strain BMG5.1 genome was analyzed via the Integrated Microbial Genomes (IMGer) platform of the Joint Genome Institute, Walnut Creek, CA. This whole genome shotgun sequence has been deposited at DDBJ/EMBL/GenBank under accession number JWIO00000000. The version described in this paper is JWIO01000000.

The draft genome was compared to that of *Candidatus* Frankia datiscae to identify those genes different between the two genomes using the default values of 30% amino acid identity over 80% of the length of the shortest sequence on the IMGer platform^33^.

### Phylogenetic analysis

Selected MLSA phylogenetic markers^34^ were downloaded from the JGI-IMG (http://img.jgi.doe.gov/cgi-bin/w/main.cgi) and used for Maximum Likelihood^35^ phylogenetic inference using MEGA 5.0^36^.

## Additional Information

**How to cite this article**: Gtari, M. *et al.* Cultivating the uncultured: growing the recalcitrant cluster-2 *Frankia* strains. *Sci. Rep.* **5**, 13112; doi: 10.1038/srep13112 (2015).

## Supplementary Material

Supplementary Information

## Figures and Tables

**Figure 1:**
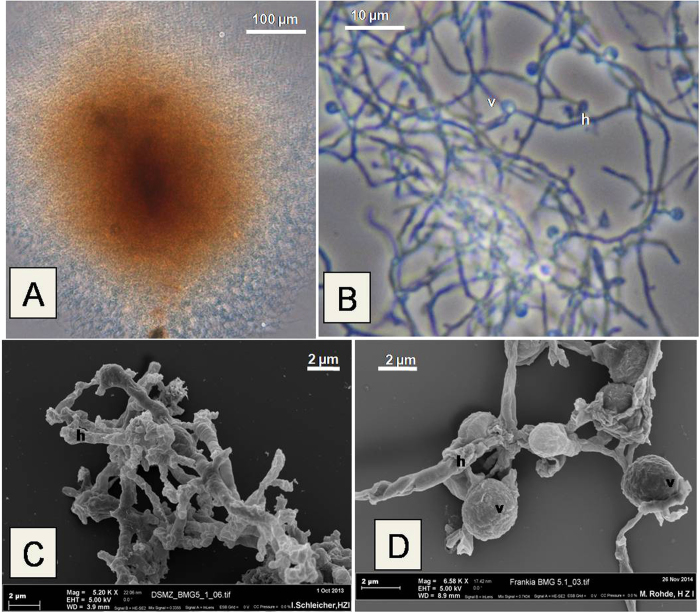
*Frankia* strain BMG5.1 in pure culture. (**A**) a micro-colony grown on BAP medium at pH 9. (**B**) a higher magnification with short hyphae (h) and spherical nitrogen-fixing vesicles (v). (**C**) and (**D**) scanning electron micrographs showing short thick hyphae and vesicles, respectively.

**Figure 2:**
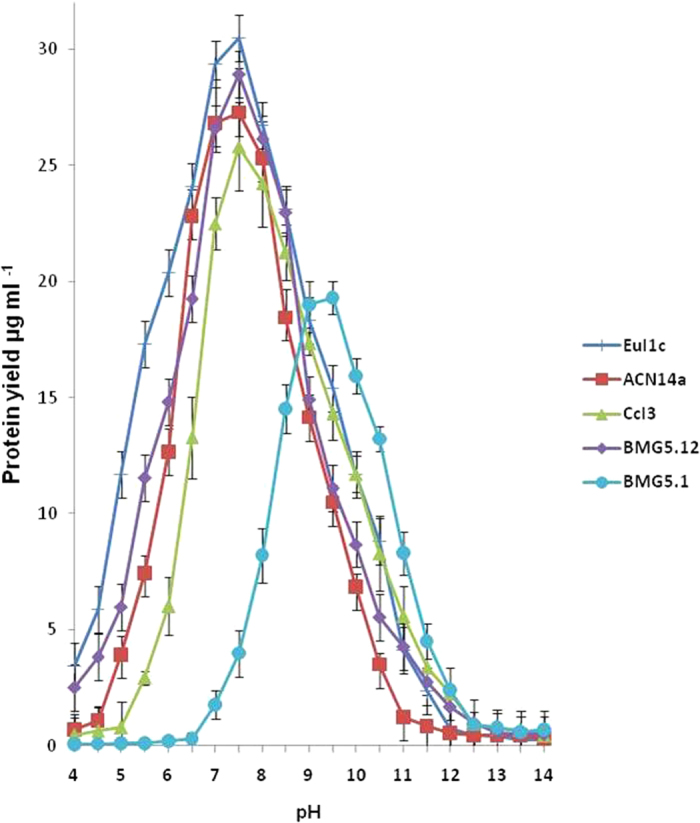
Protein yield of *Frankia* sp. strain BMG5.1 compared to *Frankia* sp. strains EuI1c (cluster-4), ACN14a (cluster-1a), CcI3 (cluster-1c) and BMG5.12 (cluster-3) grown in BAP medium with 5mM NH_4_Cl, buffered at different pH values.

**Figure 3:**
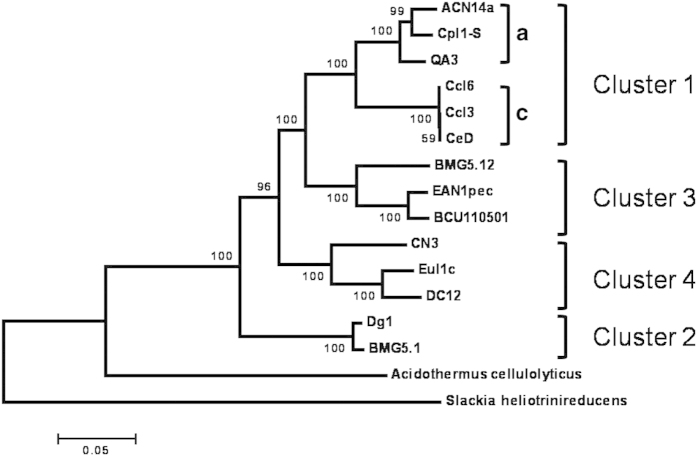
Maximum Likelihood phylogenetic tree estimated from concatenation of *atp*1, *fts*Z, *dna*K, *gyr*A and *sec*A genes. The tree was rooted using *Acidothermus cellulolyticus* and the deeply branching actinobacterium *Slackia helionitrireducens* and bootstrap values based on 1,000 replicates shown as percentages at branching points. *Frankia* clusters are designated as described previously^7^.

**Figure 4:**
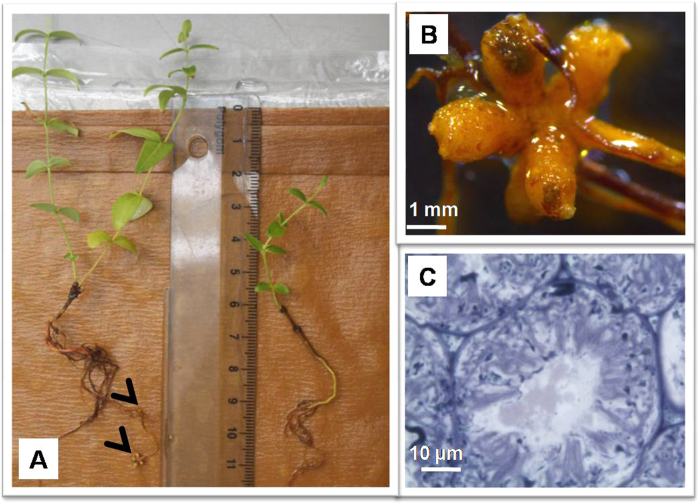
*Coriaria myrtifolia* seedlings grown in growth pouches. (**A**) seedlings were inoculated (left) with a pure culture of *Frankia* strain BMG5.1 or not inoculated (right). (**B**) a multi-lobed nodule. (**C**) a section through a cortical cell with peripheral nitrogen-fixing *Frankia* vesicles.

**Table 1:** Summary of genome characteristics of isolated *Frankia* strain BMG5.1 and the *Candidatus* Frankia datiscae Dg1.

	BMG5.1	Dg1
Status	Draft (116 scaffolds)	Finished (1 contig)
Size in bp	5,795,263	5,323,186
Proteins	5,245	4,527
Pseudogenes	375	355
rRNA	3	6
tRNA	47	44
%G+C	70.16	70.04
Transposons and IS elements	29	180
Horizontally Transferred Genes	44	1
Accession	JWIO01000000	CP002801.1

## References

[b1] Koch R. Die Aetiologie der Tuberculose. Berliner klinische Wochenschrift 19, 221–230 (1882).

[b2] Relman D. A. The identification of uncultured microbial pathogens. The Journal of infectious diseases 168, 1–8 (1993). 7685802 10.1093/infdis/168.1.1

[b3] Stewart E. J. Growing unculturable bacteria. Journal of bacteriology 194, 4151–4160, 10.1128/JB.00345-12 (2012). 22661685 PMC3416243

[b4] Woronin M. S. Uber die bei der Schwarzerle (*Alnus glutinosa*) und bei der gewöhnlichen Garten-Lupine (*Lupinus mutabilis*) auftretenden Wurzelanschwellungen. Mem Acad Imp Sci St.Petersbourg **VII Series**. 10, 1–13 (1866).

[b5] Becking J. H. Frankiaceae *fam. nov.* (Actinomycetales) with one new combination and six new species of the genus *Frankia* Brunchorst 1886, 174. Int J Syst Bacteriol 20, 201–220 (1970).

[b6] Callaham D., Del Tredici P. & Torrey J. G. Isolation and cultivation *in vitro* of the actinomycete causing root nodulation in *Comptonia*. Science 199, 899–902 (1978). 17757592 10.1126/science.199.4331.899

[b7] Normand P. *et al.* Molecular phylogeny of the genus *Frankia* and related genera and emendation of the family *Frankiaceae*. Int J Syst Bacteriol 46, 1–9 (1996). 8573482 10.1099/00207713-46-1-1

[b8] Persson T. *et al.* Genome sequence of “*Candidatus* Frankia datiscae” Dg1, the uncultured microsymbiont from nitrogen-fixing root nodules of the dicot *Datisca glomerata*. J Bacteriol 193, 7017–7018 (2011). 22123767 10.1128/JB.06208-11 PMC3232863

[b9] Persson T. *et al.* *Candidatus* Frankia datiscae Dg1, the actinobacterial microsymbiont of *Datisca glomerata*, expresses the canonical *nod* genes *nodABC* in symbiosis with its host plant. PLoS ONE 10, e0127630, 10.1371/journal.pone.0127630 (2015). 26020781 PMC4447401

[b10] Miller J. M. & Rhoden D. L. Preliminary evaluation of Biolog, a carbon source utilization method for bacterial identification. J Clin Microbiol 29, 1143–1147 (1991). 1864931 10.1128/jcm.29.6.1143-1147.1991 PMC269959

[b11] Baker D. & O’Keefe D. A modified sucrose fractionation procedure for the isolation of frankiae from actinorhizal root nodules and soil samples. Plant Soil 78, 23–28 (1984).

[b12] Murry M., Fontaine M. & Torrey J. Growth kinetics and nitrogenase induction in *Frankia* sp. HFPArI3 grown in batch culture. Plant Soil 78, 61–78 (1984).

[b13] Tisa L., McBride M. & Ensign J. C. Studies on growth and morphology of *Frankia* strains EAN1pec, EuI1c, CpI1 and ACN1AG. Can. J. Bot. 61, 2768–2773 (1983).

[b14] Murry M., Fontaine M. & Torrey J. Growth kinetics and nitrogenase induction in *Frankia* sp. HFPArI3 grown in batch culture. Plant & Soil 78, 61–78 (1984).

[b15] Tjepkema J. D., Ormerod W. & Torrey J. G. Vesicle formation and acetylene reduction activity in *Frankia* sp. CpI1 cultured in defined nutrient media. Nature 287, 633–635 (1980).

[b16] Goris J. *et al.* DNA-DNA hybridization values and their relationship to whole-genome sequence similarities. Int J Syst Evol Microbiol 57, 81–91, 10.1099/ijs.0.64483-0 (2007). 17220447

[b17] Normand P. *et al.* Genome characteristics of facultatively symbiotic *Frankia* sp. strains reflect host range and host plant biogeography. Genome Res 17, 7–15 (2007). 17151343 10.1101/gr.5798407 PMC1716269

[b18] Stewart W., Fitzgerald & Burris, R. *In situ* studies on N_2_ fixation using the acetylene reduction technique. Proc Nat Acad Sci (USA) 58, 2071–2078 (1967). 5237501 10.1073/pnas.58.5.2071 PMC223907

[b19] Silvester W. B., Langenstein B. & Berg R. H. Do mitochondria provide the oxygen diffusion barrier in root nodules of *Coriaria* and *Datisca*? Can. J. Bot. 77, 1358–1366 (1999).

[b20] Alloisio N. *et al.* The *Frankia alni* symbiotic transcriptome. Mol Plant Microbe Interact 23, 593–607 (2010). 20367468 10.1094/MPMI-23-5-0593

[b21] Carro L. *et al.* *Alnus* peptides modify membrane porosity and induce the release of nitrogen-rich metabolites from nitrogen-fixing *Frankia*. The ISME journal **10.1038/ismej. 2014**.**257**, 10.1038/ismej.2014.257 (2015). PMC4511928 25603394

[b22] Wang H. F. *et al.* *Nesterenkonia rhizosphaerae* sp. nov., a novel alkaliphilic actinobacterium isolated from rhizosphere soil of *Reaumuria soongorica* in saline-alkaline desert. Int J Syst Evol Microbiol, 10.1099/ijs.0.066894-0 (2014). 25225260

[b23] Nouioui I. *et al.* Absence of cospeciation between the uncultured *Frankia* microsymbionts and the disjunct actinorhizal Coriaria *species*. BioMed research international 2014, 924235, 10.1155/2014/924235 (2014). 24864264 PMC4016943

[b24] Benson D. Isolation of *Frankia* strains from alder actinorhizal root nodules. Applied and Environmental Microbiology 44, 461–465 (1982). 16346079 10.1128/aem.44.2.461-465.1982 PMC242032

[b25] Hoagland D. R. & Arnon D. I. The Water Culture Method for Growing Plants Without Soil., 142 (Circular 347, California Agricultural Experiment Station, 1950).

[b26] Daffonchio D., Borin S., Frova G., Manachini P. L. & Sorlini C. PCR fingerprinting of whole genomes: the spacers between the 16S and 23S rRNA genes and of intergenic tRNA gene regions reveal a different intraspecific genomic variability of *Bacillus cereus* and *Bacillus licheniformis* [corrected]. Int J Syst Bacteriol 48, 107–116 (1998). 9542081 10.1099/00207713-48-1-107

[b27] Gtari M. *et al.* Genetic diversity among *Elaeagnus* compatible *Frankia* strains and sympatric-related nitrogen-fixing actinobacteria revealed by *nifH* sequence analysis. Soil Biol. Biochem. 39, 372–377 (2007).

[b28] Nouioui I., Ghodhbane-Gtari F., Beauchemin N. J., Tisa L. S. & Gtari M. Phylogeny of members of the *Frankia* genus based on *gyrB, nifH* and *glnII* sequences. Antonie Van Leeuwenhoek 100, 579–587, 10.1007/s10482-011-9613-y (2011). 21713368

[b29] Clawson M. L., Bourret A. & Benson D. R. Assessing the phylogeny of *Frankia*-actinorhizal plant nitrogen-fixing root nodule symbioses with *Frankia* 16S rRNA and glutamine synthetase gene sequences. Mol Phylogenet Evol 31, 131–138 (2004). 15019614 10.1016/j.ympev.2003.08.001

[b30] Altschul S. F., Gish W., Miller W., Myers E. W. & Lipman D. J. Basic local alignment search tool. J Molec Biol 215, 403–410 (1990). 2231712 10.1016/S0022-2836(05)80360-2

[b31] Smith P. K. *et al.* Measurement of protein using bicinchoninic acid. Analytical biochemistry 150, 76–85 (1985). 3843705 10.1016/0003-2697(85)90442-7

[b32] Zerbino D. R. & Birney E. Velvet: algorithms for de novo short read assembly using de Bruijn graphs. Genome Res 18, 821–829, 10.1101/gr.074492.107 (2008). 18349386 PMC2336801

[b33] Markowitz V. M. *et al.* The integrated microbial genomes (IMG) system. Nucleic Acids Res 34, D344–348, 10.1093/nar/gkj024 (2006). 16381883 PMC1347387

[b34] Sen A. *et al.* Phylogeny of the class Actinobacteria revisited in the light of complete genomes. The orders ‘*Frankiales*’ and *Micrococcales* should be split into coherent entities: proposal of *Frankiales* ord. nov., *Geodermatophilales* ord. nov., *Acidothermales* ord. nov. and *Nakamurellales* ord. nov. Int J Syst Evol Microbiol 64, 3821–3832, 10.1099/ijs.0.063966-0 (2014). 25168610

[b35] Felsenstein J. Evolutionary trees from DNA sequences: A maximum likelihood approach. J. Mol. Evol. 17, 368–376 (1981). 7288891 10.1007/BF01734359

[b36] Tamura K., Stecher G., Peterson D., Filipski A. & Kumar S. MEGA6: Molecular Evolutionary Genetics Analysis version 6.0. Mol Biol Evol 30, 2725–2729, 10.1093/molbev/mst197 (2013). 24132122 PMC3840312

