# Supplementary material for: Cultivating the uncultured: growing the recalcitrant cluster-2 Frankia strains
Source: Sci Rep. 2015 Aug 19;5:13112. doi: 10.1038/srep13112 (PMC4541404; doi:10.1038/srep13112)
Supplement: Supplementary Information [file srep13112-s1.doc]

Supplementary Materials_Cultivating the uncultured: growing the recalcitrant cluster-2 *Frankia* strains

Maher Gtari^1*^, Faten Ghodhbane-Gtari^1^, Imen Nouioui^1^, Amir Ktari^1^, Karima Hezbri^1^, Wajdi Mimouni^1^, Imed Sbissi^1^, Amani Ayari^1^, Takashi Yamanaka^2^, Philippe Normand^3^, Louis S Tisa^4^, Abdellatif Boudabous^1^

^1^ Laboratoire Microorganismes et Biomolécules Actives, Université de Tunis El Manar (FST) & Université Carthage (INSAT), 2092, Tunis, Tunisia.

^2^ Forest and Forestry Products Research Institute, Ibaraki 305-8687, Japan.

^3^ Ecologie microbienne, UMR CNRS 5557, Université Lyon1, Université de Lyon, 69622 cedex, Villeurbanne, France.

^4^ Department of Molecular, Cellular and Biomedical Sciences, University of New Hampshire, Durham, NH 03824, USA.

Supplementary Figure and Tables

Fig. S1- Abundance of antiporter genes in BMG5.1 strain and related *Frankia* genomes.

Supplementary Tables:

Table S1-Phenoarray of BMG5.1 strain using Biolog microplates for carbon sources (PM1 and PM2A), pH (PM10) and osmolytes (PM9). Microplates were incubated at 25°C for 4 weeks. The values are arbitrary units yielded by the Biolog plate reader. The values given in table1 are the average of two biological replicates with a standard deviation ≤5 %. Those values below the negative controls (49) were considered negative.

| PM1 microplate | | | PM2A microplate | | | | PM10 microplate | | | PM9 microplate | |  |
| --- | --- | --- | --- | --- | --- | --- | --- | --- | --- | --- | --- | --- |
| A1 | Negative control | 49 | A1 |  | Negative control | 49 | A1 | pH3.5 | 31 | A1 | NaCl 1% | 195 |
| A2 | L- Arabinose | 105 | A2 |  | Chondroitin Sulfate C | 37 | A2 | pH4 | 32 | A2 | NaCl 2% | 170 |
| A3 | N-Acetyl-D-Glucosamine | 97 | A3 |  | α-Cyclodextrin | 28 | A3 | pH4.5 | 32 | A3 | NaCl 3% | 44 |
| A4 | D-Saccharic Acid | 33 | A4 |  | β-Cyclodextrin | 38 | A4 | pH5 | 35 | A4 | NaCl 4% | 42 |
| A5 | Succinic Acid | 199 | A5 |  | γ-Cyclodextrin | 30 | A5 | pH5.5 | 46 | A5 | NaCl 5 % | 33 |
| A6 | D-Galactose | 41 | A6 |  | Dextrin | 32 | A6 | pH6 | 51 | A6 | NaCl 5.5% | 42 |
| A7 | L-Aspartic Acid | 136 | A7 |  | Gelatin | 47 | A7 | pH7 | 64 | A7 | NaCl 6% | 26 |
| A8 | L-Proline | 31 | A8 |  | Glycogen | 46 | A8 | pH8 | 97 | A8 | NaCl 6.5% | 37 |
| A9 | D-Alanine | 53 | A9 |  | Inulin | 49 | A9 | pH8.5 | 127 | A9 | NaCl 7% | 35 |
| A10 | D-Threhalose | 60 | A10 |  | Laminarin | 43 | A10 | pH9 | 193 | A10 | NaCl 8% | 34 |
| A11 | D-Mannose | 51 | A11 |  | Mannan | 55 | A11 | pH9.5 | 169 | A11 | NaCl 9% | 42 |
| A12 | Dulcitol | 52 | A12 |  | Pectin | 88 | A12 | pH10 | 133 | A12 | NaCl 10% | 50 |
| B1 | D-Serine | 62 | B1 |  | N-Acetyl-D- Galactosamine | 39 | B1 | pH4.5 | 41 | B1 | NaCl 6% | 51 |
| B2 | D-Sorbitol | 34 | B2 |  | N-Acetyl-Neuraminic Acid | 20 | B2 | pH4.5 + L-Alanine | 13 | B2 | NaCl 6% + Betaine | 93 |
| B3 | Glycerol | 33 | B3 |  | β-D-Allose | 33 | B3 | pH4.5 + L-Arginine | 30 | B3 | NaCl 6% +N-N Dimethyl Glycine | 41 |
| B4 | L-Fructose | 30 | B4 |  | Amyddalin | 17 | B4 | pH5.4+ L-Asparagine | 34 | B4 | NaCl 6% Sarcosine | 35 |
| B5 | D-Glucuronic Acid | 81 | B5 |  | D-Arabinose | 184 | B5 | pH4.5 + L-Aspartic Acid | 37 | B5 | NaCl 6% Dimethyl sulphonyl propionate | 48 |
| B6 | D-Gluconic Acid | 24 | B6 |  | D-Arabitol | 38 | B6 | pH4.5 + L-Glutamic Acid | 32 | B6 | NaCl 6% + 3-(N-morpholino)propanesulfonic acid | 37 |
| B7 | D,L-α-Glycerol-Phosphate | 51 | B7 |  | L-Arabitol | 31 | B7 | pH4.5 + L-Glutamine | 44 | B7 | NaCl 6% + Ectoine | 34 |
| B8 | D-Xylose | 122 | B8 |  | Arbutin | 20 | B8 | pH4.5 + LGlycine | 36 | B8 | NaCl 6% +Choline | 37 |
| B9 | L-Lactic Acid | 76 | B9 |  | 2-Deoxy-D-Ribose | 196 | B9 | pH4.5 + L-Histidine | 57 | B9 | NaCl 6% +Phosphoryl Choline | 45 |
| B10 | Formic Acid | 62 | B10 |  | i-Erythritol | 34 | B10 | pH4.5 + L-Isoleucine | 74 | B10 | NaCl 6% + Cratine | 51 |
| B11 | D-Mannitol | 39 | B11 |  | D-Fucose | 41 | B11 | pH4.5 + L- Leucine | 47 | B11 | NaCl 6%+ Creatinine | 125 |
| B12 | L-Glutamic Acid | 164 | B12 |  | L-3-0-β-D-Galacto-Pyranosyl-D-Arabinose | 47 | B12 | pH4.5 + L-Lysine | 60 | B12 | NaCl 6% + Carnitine | 51 |
| C1 | D-Glucose-6-Phosphate | 35 | C1 |  | Gentiobiose | 24 | C1 | pH4.5 + L-Methionine | 44 | C1 | NaCl 6% +KCl | 47 |
| C2 | D-Galactonic Acid-γ--Lactone | 116 | C2 |  | L-Glucose | 23 | C2 | pH4.5 + L-Phenylalanine | 42 | C2 | NaCl 6% + L-Proline | 106 |
| C3 | D, L-Malic Acid | 23 | C3 |  | Lactitol | 18 | C3 | pH4.5 + L-Proline | 45 | C3 | NaCl 6% + N-Acetyl L-Glutamine | 45 |
| C4 | D-Ribose | 186 | C4 |  | D-Melezitose | 21 | C4 | pH4.5 + L-Serine | 29 | C4 | NaCl 6%+ β-Glutamic Acid | 36 |
| C5 | Tween 20 | 35 | C5 |  | TMaltitol | 31 | C5 | pH4.5 + L-Threonine | 37 | C5 | NaCl 6% + γ-Amino-N- Butyric Acid | 55 |
| C6 | L-Rhamnose | 79 | C6 |  | a-Methyl-D-Glucoside | 31 | C6 | pH4.5 + L-Tryptophane | 81 | C6 | NaCl 6% +Glutatione | 61 |
| C7 | D-Fructose | 29 | C7 |  | β-Methyl-D-Galactoside | 24 | C7 | pH4.5 + L-Citrulline | 41 | C7 | NaCl 6%+ Glycerol | 37 |
| C8 | Acetic Acid | 198 | C8 |  | 3-Methyl Glucose | 29 | C8 | pH4.5 + L-Valine | 42 | C8 | NaCl 6% + Trehalose | 45 |
| C9 | α-D-Glucose | 55 | C9 |  | β-Methyl-D-Glucuronic Acid | 34 | C9 | pH4.5 + L- Hydroxy-L-Proline | 50 | C9 | NaCL 6% + Trimethylamine-N-oxide | 52 |
| C10 | Maltose | 37 | C10 |  | α-Methyl-D-Mannoside | 40 | C10 | pH4.5 + L-Ornithine | 42 | C10 | NaCl 6% + Trimethylamine | 116 |
| C11 | D-Melibiose | 57 | C11 |  | β-Methyl-D-Xyloside | 36 | C11 | pH4.5 + L-Homoarginine | 48 | C11 | NaCl 6% Octopine | 54 |
| C12 | Thymidine | 146 | C12 |  | Palatinose | 67 | C12 | pH4.5 + L-Homoserine | 73 | C12 | NaCl 6% Trigonelline | 51 |
| D1 | L-Asparagine | 83 | D1 |  | D-Raffinose | 23 | D1 | pH4.5 + Anthranilic Acid | 66 | D1 | KCl 3% | 96 |
| D2 | D-Aspartic Acid | 51 | D2 |  | Salicin | 14 | D2 | pH4.5 + L-Noleucine | 86 | D2 | KCl 4% | 99 |
| D3 | D-Glucosaminic Acid | 29 | D3 |  | Sedoheptulosan | 73 | D3 | pH4.5 + L-Norvaline | 62 | D3 | KCl 5% | 55 |
| D4 | 1,2-Propanediol | 32 | D4 |  | L-Sorbose | 33 | D4 | pH4.5 + α- Amino-N- Butyric Acid | 44 | D4 | KCl 6% | 63 |
| D5 | Tween 40 | 34 | D5 |  | Stachyose | 38 | D5 | pH4.5 + p-Amino- Benzoic Acid | 62 | D5 | Sodium sulfate 2% | 178 |
| D6 | α-Keto-Glutaric Acid | 83 | D6 |  | D-Tagatose | 155 | D6 | pH4.5 + L-Cysteic Acid | 49 | D6 | Sodium sulfate 3% | 150 |
| D7 | α-Keto-Butyric Acid | 36 | D7 |  | Turanose | 75 | D7 | pH4.5 + D-Lysine | 42 | D7 | Sodium sulfate 4% | 121 |
| D8 | α-Methyl-D-Galactoside | 31 | D8 |  | Xylitol | 32 | D8 | pH4.5 + 5-Hydroxy Lysine | 37 | D8 | Sodium sulfate 5% | 104 |
| D9 | α-D-Lactose | 65 | D9 |  | N-Acetyl-D-Glucosaminitol | 28 | D9 | pH4.5 + 5-Hydroxy Tryptophan | 62 | D9 | Ethylene glycol 5% | 101 |
| D10 | Lactulose | 100 | D10 |  | γ-Amino Butyric Acid | 44 | D10 | pH4.5 + D, L-Diaminopimelic Acid | 45 | D10 | Ethylene glycol 10% | 89 |
| D11 | Sucrose | 99 | D11 |  | δ-Amino Valeric Acid | 57 | D11 | pH4.5 + Trimethylamine-N-oxide | 51 | D11 | Ethylene glycol 15% | 57 |
| D12 | Uridine | 198 | D12 |  | Butyric Acid | 38 | D12 | pH4.5 + Urea | 62 | D12 | Ethylene glycol 20% | 58 |
| E1 | L-Glutamine | 86 | E1 |  | Capric Acid | 22 | E1 | pH9.5 + L-Alanine | 74 | E1 | Sodium formate 1% | 107 |
| E2 | m-Tartric Acid | 31 | E2 |  | Capronic Acid | 18 | E2 | pH9.5 + L-Arginine | 69 | E2 | Sodium formate 2% | 72 |
| E3 | D-Glucose-1-Phosphate | 76 | E3 |  | Citraconic Acid | 15 | E3 | pH5.4+ L-Asparagine | 50 | E3 | Sodium formate 3% | 47 |
| E4 | D-Fructose-6-Phosphate | 59 | E4 |  | Citramalic Acid | 14 | E4 | pH9.5 | 63 | E4 | Sodium formate 4% | 46 |
| E5 | Tween 80 | 57 | E5 |  | D-Glucosamine | 128 | E5 | pH9.5 + L-Aspartic Acid | 157 | E5 | Sodium formate 5% | 73 |
| E6 | α-HydroxyGlutaric Acid-γ-Lactone | 69 | E6 |  | 2-Hydroxy Benzoic Acid | 18 | E6 | pH9.5 + L-Glutamic Acid | 121 | E6 | Sodium formate 6% | 58 |
| E7 | α-Hydroxy Butyric Acid | 101 | E7 |  | 4-Hydroxy Butyric Acid | 15 | E7 | pH9.5 + L-Glutamine | 114 | E7 | Urea 2% | 109 |
| E8 | β-Methyl-D- Glucoside | 29 | E8 |  | β-Hydroxy Butyric Acid | 21 | E8 | pH9.5 + LGlycine | 213 | E8 | Urea 3% | 108 |
| E9 | Adonitol | 58 | E9 |  | γ-Hydroxy Butyric Acid | 32 | E9 | pH9.5 + L-Histidine | 178 | E9 | Urea 4% | 105 |
| E10 | Maltotriose | 49 | E10 |  | a-Keto-Valeric Acid | 26 | E10 | pH9.5 + L-Isoleucine | 118 | E10 | Urea 5% | 50 |
| E11 | 2-Deoxy Adenosine | 51 | E11 |  | 2-Deoxy AdItaconicAcidenosine | 32 | E11 | pH9.5 + L- Leucine | 124 | E11 | Urea 6% | 53 |
| E12 | Adenosine | 81 | E12 |  | 5-Keto-D- Gluconic Acid | 98 | E12 | pH9.5 + L-Lysine | 178 | E12 | Urea 7% | 52 |
| F1 | Glycyl-L-Aspartic Acid | 55 | F1 |  | D-Lactic Acid Methyl Ester | 25 | F1 | pH9.5 + L-Methionine | 115 | F1 | Sodium lactate 1% | 204 |
| F2 | Citric Acid | 173 | F2 |  | Malonic Acid | 24 | F2 | pH9.5 + L-Phenylalanine | 166 | F2 | Sodium lactate 2% | 200 |
| F3 | m-Inositol | 39 | F3 |  | Melibionic Acid | 22 | F3 | pH9.5 + L-Proline | 165 | F3 | Sodium lactate 3% | 101 |
| F4 | D-Threonine | 40 | F4 |  | Oxalic Acid | 21 | F4 | pH9.5 + L-Serine | 206 | F4 | Sodium lactate 4% | 72 |
| F5 | Fumaric Acid | 96 | F5 |  | Oxalomalic Acid | 34 | F5 | pH9.5 + L-Threonine | 154 | F5 | Sodium lactate 5% | 71 |
| F6 | Bromo Succinic Acid | 38 | F6 |  | Quinic Acid | 15 | F6 | pH9.5 + L-Tryptophane | 207 | F6 | Sodium lactate 6% | 49 |
| F7 | Propionic Acid | 215 | F7 |  | D-Ribono-1-4-Lactone | 18 | F7 | pH9.5 + L-Tyrosine | 233 | F7 | Sodium lactate 7% | 44 |
| F8 | Mucic Acid | 38 | F8 |  | Sebacic Acid | 24 | F8 | pH9.5 + L-Valine | 127 | F8 | Sodium lactate 8% | 40 |
| F9 | Glycolic Acid | 51 | F9 |  | Sorbic Acid | 102 | F9 | pH9.5 + L- Hydroxy-L-Proline | 109 | F9 | Sodium lactate 9% | 43 |
| E10 | Glyoxylic Acid | 68 | E10 |  | Succinamic Acid | 30 | E10 | pH9.5 + L-Ornithine | 127 | E10 | Sodium lactate 10% | 42 |
| F11 | D-Cellobiose | 72 | F11 |  | D-Tartric Acid | 47 | F11 | pH9.5 + L-Homoarginine | 152 | F11 | Sodium lactate 11% | 40 |
| F12 | Inosine | 72 | F12 |  | L-Tartric Acid | 40 | F12 | pH9.5 + L-Homoserine | 174 | F12 | Sodium lactate 12% | 41 |
| G1 | Glycyl-L-Glutamic Acid | 39 | G1 |  | Acetamide | 26 | G1 | pH9.5 + Anthranilic Acid | 182 | G1 | Sodium Phosphate pH7 20mM | 46 |
| G2 | Tricarballylic Acid | 115 | G2 |  | L-Alaninamide | 16 | G2 | pH9.5 + L-Noleucine | 139 | G2 | Sodium Phosphate pH7 50mM | 46 |
| G3 | L-Serine | 31 | G3 |  | N-Acetyl-L-Glutamic Acid | 22 | G3 | pH9.5 + L-Norvaline | 104 | G3 | Sodium Phosphate pH7 100mM | 47 |
| G4 | L-Threonine | 90 | G4 |  | L-Arginine | 25 | G4 | pH9.5 + Arginine | 105 | G4 | Sodium Phosphate pH7 200mM | 48 |
| G5 | L-Alanine | 32 | G5 |  | Glycine | 21 | G5 | pH9.5 + Cadaverine | 180 | G5 | Sodium Benzoate pH5.2 20mM | 40 |
| G6 | L-Alanyl-Glycine | 77 | G6 |  | L-Histidine | 28 | G6 | pH9.5 + Putrescine | 125 | G6 | Sodium Benzoate pH5.2 50mM | 49 |
| G7 | Acetoacetic Acid | 122 | G7 |  | L-Homoserine | 40 | G7 | pH9.5 + Histamine | 242 | G7 | Sodium Benzoate pH5.2 100mM | 47 |
| G8 | N-Acetyl-β-D-Mannosamine | 59 | G8 |  | Hydroxy-L-Proline | 30 | G8 | pH9.5 + Phenylalanine | 132 | G8 | Sodium Benzoate pH5.2 200mM | 43 |
| G9 | Mono Methyl Succinate | 114 | G9 |  | L-Isoleucine | 23 | G9 | pH9.5 + Tyramine | 199 | G9 | Ammonium sulfate pH8 10mM | 153 |
| G10 | Methyl Pyruvate | 57 | G10 |  | L-Leucine | 40 | G10 | pH9.5 + Creatine | 115 | G10 | Ammonium sulfate pH8 20mM | 145 |
| G11 | D-Malic Acid | 73 | G11 |  | L-Lysine | 51 | G11 | pH9.5 + Trimethylamine-N-oxide | 119 | G11 | Ammonium sulfate pH8 50mM | 139 |
| G12 | L-Malic Acid | 102 | G12 |  | L-Methionine | 43 | G12 | pH9.5 + Urea | 127 | G12 | Ammonium sulfate pH8 100mM | 69 |
| H1 | Glycyl-L-Proline | 167 | H1 |  | L-Ornithine | 45 | H1 | X-Caprylate | 111 | H1 | Sodium Nitrate 10mM | 104 |
| H2 | p-Hydroxy Phenyl Acetic Acid | 157 | H2 |  | L-Phenylalanine | 28 | H2 | X-α-D-Glucoside | 73 | H2 | Sodium Nitrate 20mM | 101 |
| H3 | m-Hydroxy Phenyl Acetic Acid | 73 | H3 |  | L-Pyroglutamic Acid | 21 | H3 | X-β-D-Glucoside | 58 | H3 | Sodium Nitrate 40mM | 105 |
| H4 | Tyramine | 62 | H4 |  | L-Valine | 25 | H4 | X-α-D-Galactoside | 68 | H4 | Sodium Nitrate 60mM | 106 |
| H5 | D-Psicose | 59 | H5 |  | D, L-Carnitine | 20 | H5 | X-β-D-Galactoside | 68 | H5 | Sodium Nitrate 80mM | 105 |
| H6 | L-Lyxose | 123 | H6 |  | Sec-Butylamine | 19 | H6 | X-α-D- Glucuronide | 75 | H6 | Sodium Nitrate 100mM | 103 |
| H7 | Glucuronamide | 78 | H7 |  | D, L-Octopamine | 31 | H7 | X-β-D- Glucuronide | 74 | H7 | Sodium Nitrite 10mM | 105 |
| H8 | Pyruvic Acid | 243 | H8 |  | Putrescine | 28 | H8 | X-β-D-glucosaminide | 82 | H8 | Sodium Nitrite 20mM | 106 |
| H9 | L-Galacturonic Acid | 84 | H9 |  | Dhydroxy Acetone | 112 | H9 | X-β-D- Galactosaminide | 83 | H9 | Sodium Nitrite 40mM | 84 |
| H10 | D-Galacturonic Acid | 80 | H10 |  | 2,3-Butanediol | 45 | H10 | X-α-D- Mannoside | 109 | H10 | Sodium Nitrite 60mM | 85 |
| H11 | Phenylethyl-Amine | 116 | H11 |  | 2,3-Butanone | 86 | H11 | X-PO4 | 88 | H11 | Sodium Nitrite 80mM | 82 |
| H12 | 2-Aminoethanol | 115 | H12 |  | 3-Hydroxy 2-Butanone | 55 | H12 | X-SO4 | 89 | H12 | Sodium Nitrite 100mM | 85 |

Tables S2-Gene content differences between cluster-2 *Frankia* strain BMG5.1 and *Candidatus* Frankia datiscae Dg1.

Genes present in BMG5.1 and absent in Dg1

| Gategory | Role |
| --- | --- |
| AminoAcids and Derivatives | N-carbamoylputrescine amidase (3.5.1.53) |
| AminoAcids and Derivatives | Threonine dehydratase (EC 4.3.1.19) |
| AminoAcids and Derivatives | 5'-methylthioadenosine phosphorylase (EC 2.4.2.28) |
| AminoAcids and Derivatives | Threonine dehydrogenase and related Zn-dependentdehydrogenases |
| AminoAcids and Derivatives | L-Proline/Glycine betaine transporter ProP |
| Carbohydrates | Glycolatedehydrogenase (EC 1.1.99.14), subunitGlcD |
| Carbohydrates | Putative phosphoenolpyruvate synthase/pyruvate phosphate dikinase, C-terminal domain |
| Carbohydrates | 2-methylcitrate synthase (EC 2.3.3.5) |
| Cell Wall and Capsule | GDP-mannose 4,6-dehydratase (EC 4.2.1.47) |
| Cell Wall and Capsule | Lipoprotein releasing system ATP-binding protein LolD |
| Clustering-basedsubsystems | Hydroxyacylglutathione hydrolase (EC 3.1.2.6) |
| Clustering-basedsubsystems | Histone acetyltransferase HPA2 and related acetyltransferases |
| Clustering-basedsubsystems | Adenine-specific methyl transferase (EC 2.1.1.72) |
| Clustering-basedsubsystems | Alpha-1,3-N-acetylgalactosamine transferasePglA (EC 2.4.1.-) |
| Clustering-basedsubsystems | Lipid carrier : UDP-N-acetylgalactosaminyl transferase (EC 2.4.1.-) |
| Clustering-basedsubsystems | Nucleoside-diphosphate-sugar epimerases |
| Clustering-basedsubsystems | Putative glycosyltransferase |
| Cofactors, Vitamins, Prosthetic Groups, Pigments | Butyryl-CoAdehydrogenase (EC 1.3.8.1) |
| Cofactors, Vitamins, Prosthetic Groups, Pigments | 2-amino-4-hydroxy-6-hydroxymethyldihydropteridine pyrophosphokinase (EC 2.7.6.3) |
| Cofactors, Vitamins, Prosthetic Groups, Pigments | Periplasmic molybdate-binding domain |
| DNA Metabolism | CRISPR-associated RecB family exonuclease Cas4a |
| DNA Metabolism | CRISPR-associated negative autoregulator, Cst2 family |
| DNA Metabolism | CRISPR-associated protein Cas1 |
| DNA Metabolism | CRISPR-associated protein Cas2 |
| DNA Metabolism | CRISPR-associated protein, Cas5t family |
| DNA Metabolism | CRISPR-associated RAMP Cmr1 |
| DNA Metabolism | CRISPR-associated RAMP Cmr2 |
| DNA Metabolism | CRISPR-associated RAMP Cmr3 |
| DNA Metabolism | CRISPR-associated RAMP Cmr4 |
| DNA Metabolism | CRISPR-associated RAMP Cmr6 |
| DNA Metabolism | ATP-dependent nuclease, subunit A |
| DNA Metabolism | ATP-dependent nuclease, subunit B |
| DNA Metabolism | ATP-dependent DNA ligase (EC 6.5.1.1) |
| Fatty Acids, Lipids, and Isoprenoids | 3-oxoacyl-[acyl-carrier-protein] synthase, KASIII (EC 2.3.1.41) |
| Fatty Acids, Lipids, and Isoprenoids | Geranylgeranyldiphosphate synthase (EC 2.5.1.29) |
| Fatty Acids, Lipids, and Isoprenoids | Hydroxymethylglutaryl-CoA synthase (EC 2.3.3.10) |
| Fatty Acids, Lipids, and Isoprenoids | D-beta-hydroxybutyratedehydrogenase (EC 1.1.1.30) |
| Iron acquisition and metabolism | 2,3-dihydroxybenzoate-AMP ligase (EC 2.7.7.58) of siderophore biosynthesis |
| Iron acquisition and metabolism | Putative ABC iron siderophore transporter, fused permease and ATPase domains |
| Membrane Transport | Oligopeptide transport ATP-binding protein OppD (TC 3.A.1.5.1) |
| Membrane transport | Formate hydrogenlyase subunit 3/Multisubunit Na+/H+ antiporter, MnhD subunit |
| Membrane transport | multisubunit sodium/proton antiporter, MrpG subunit (TC 2.A.63.1) |
| Membrane transport | multisubunit sodium/proton antiporter, MrpE subunit (TC 2.A.63.1) |
| Membrane transport | Na+/H+ antiporter NhaD and related arsenite permeases |
| Membrane transport | potassium/proton antiporter membrane subunit, CPA2 family (TC 2.A.37.5.2) |
| Metabolism of Aromatic Compounds | Gentisate 1,2-dioxygenase (EC 1.13.11.4) |
| NitrogenMetabolism | Assimilatory nitrate reductase large subunit (EC:1.7.99.4) |
| NitrogenMetabolism | Nitrate/nitrite transporter |
| NitrogenMetabolism | Nitrite reductase [NAD(P)H] large subunit (EC 1.7.1.4) |
| NitrogenMetabolism | Nitrite reductase [NAD(P)H] small subunit (EC 1.7.1.4) |
| Nucleosides and Nucleotides | Phosphoribosyl formylglycinamidine cyclo-ligase (EC 6.3.3.1) |
| Nucleosides and Nucleotides | Thymidine kinase (EC 2.7.1.21) |
| Nucleosides and Nucleotides | Ribonucleotidereductase of class III (anaerobic), activating protein (EC 1.97.1.4) |
| Nucleosides and Nucleotides | Ribonucleotidereductase of class Ia (aerobic), beta subunit (EC 1.17.4.1) |
| Phages, Prophages, Transposable elements, Plasmids | Phage major capsid protein |
| Phages, Prophages, Transposable elements, Plasmids | Phage portal protein |
| Phages, Prophages, Transposable elements, Plasmids | Phage terminase large subunit |
| Phages, Prophages, Transposable elements, Plasmids | Phage terminase, small subunit |
| Phages, Prophages, Transposable elements, Plasmids | DNA primase/helicase, phage-associated |
| Phages, Prophages, Transposable elements, Plasmids | Phage tail length tape-measure protein |
| PhosphorusMetabolism | secretedalkaline phosphatase |
| ProteinMetabolism | Survival protein SurA precursor (Peptidyl-prolyl cis-transisomeraseSurA) (EC 5.2.1.8) |
| ProteinMetabolism | Urease alpha subunit (EC 3.5.1.5) |
| ProteinMetabolism | intein-containing |
| RNA Metabolism | ATP-dependent RNA helicase RhlE |
| Regulation and Cellsignaling | VapC toxin protein antagonist |
| Regulation and Cellsignaling | Sporulation regulatory protein WhiD |
| SecondaryMetabolism | Lanthionine biosynthesis cyclase LanC |
| SecondaryMetabolism | Lanthionine biosynthesis protein LanB |
| SecondaryMetabolism | O-methyltransferase clustered with LanBC |
| Stress Response | Choline dehydrogenase (EC 1.1.99.1) |
| Stress Response | Aquaporin Z |
| Stress Response | Glutathione synthetase (EC 6.3.2.3) |
| Stress Response | Glutathione S-transferase, omega (EC 2.5.1.18) |
| Stress Response | Catalase (EC 1.11.1.6) |
| Stress Response | NADPH:quinoneoxidoreductase 2 |
| SulfurMetabolism | Putative arylsulfatase regulatory protein |

Genes present in Dg1 and absent in BMG5.1

| Gategory | Role |
| --- | --- |
| Amino Acids and Derivatives | Proline iminopeptidase (EC 3.4.11.5) |
| Carbohydrates | Trehalose synthase (EC 5.4.99.16) |
| Carbohydrates | Ribokinase (EC 2.7.1.15) |
| Cell Wall and Capsule | Glucose-1-phosphate thymidylyltransferase (EC 2.7.7.24) |
| Cell Wall and Capsule | UDP-N-acetylmuramoylalanine--D-glutamate ligase (EC 6.3.2.9) |
| Clustering-based subsystems | Major facilitator superfamily (MFS) transporter |
| Clustering-based subsystems | Acyl dehydratase |
| Clustering-based subsystems | MaoC domain protein dehydratase |
| Clustering-based subsystems | Sulfatase modifying factor 1 precursor (C-alpha-formylglycine- generating enzyme 1) |
| Clustering-based subsystems | tRNApseudouridine synthase B (EC 4.2.1.70) |
| Clustering-based subsystems | FIG019045: long form Mg-chelase associated protein with vWA domain |
| Clustering-based subsystems | FMN adenylyltransferase (EC 2.7.7.2) |
| Clustering-based subsystems | Riboflavin kinase (EC 2.7.1.26) |
| Clustering-based subsystems | FIG032766: hypothetical protein |
| Clustering-based subsystems | Protein serine/threonine phosphatase PrpC, regulation of stationary phase |
| Cofactors, Vitamins, Prosthetic Groups, Pigments | Aspartate 1-decarboxylase (EC 4.1.1.11) |
| Cofactors, Vitamins, Prosthetic Groups, Pigments | Nicotinate-nucleotide adenylyltransferase (EC 2.7.7.18) |
| Iron acquisition and metabolism | Thioesterase in siderophore biosynthesis gene cluster |
| Membrane Transport | Mg/Co/Ni transporter MgtE |
| Metabolism of Aromatic Compounds | Fumarylacetoacetase (EC 3.7.1.2) |
| Metabolism of Aromatic Compounds | 4-hydroxy-2-oxovalerate aldolase (EC 4.1.3.39) |
| Miscellaneous | YbbM seven transmembrane helix protein |
| Nucleosides and Nucleotides | 5-nucleotidase SurE (EC 3.1.3.5) |
| Nucleosides and Nucleotides | Adenine phosphoribosyl transferase (EC 2.4.2.7) |
| Nucleosides and Nucleotides | Carbamoyl-phosphate synthase large chain (EC 6.3.5.5) |
| Protein Metabolism | LSU ribosomal protein L33p, zinc-independent |
| Protein Metabolism | LSU ribosomal protein L4p (L1e) |
| Protein Metabolism | SSU ribosomal protein S14p (S29e), zinc-independent |
| Protein Metabolism | SSU ribosomal protein S18p, zinc-independent |
| Protein Metabolism | Putative metal chaperone, involved in Zn homeostasis, GTPase of COG0523 family |
| Protein Metabolism | putative periplasmic protein kinase ArgK and related GTPases of G3E family |
| Protein Metabolism | Serine/threonine-protein kinase RIO1 (EC 2.7.11.1) |
| RNA Metabolism | tRNA-i(6)A37 methylthiotransferase |
| RNA Metabolism | Ribonuclease E (EC 3.1.26.12) |
| RNA Metabolism | Ribonuclease D (EC 3.1.26.3) |
| Respiration | NADH-ubiquinone oxidoreductase chain H (EC 1.6.5.3) |
| Stress Response | Outer membrane protein A precursor |
| Sulfur Metabolism | sulfonate monooxygenase |
